# PHYSICAL AND SOCIAL ACTIVITIES AND COGNITIVE CHANGES AMONG OLD-OLD ADULTS: RESULTS FROM 3-YEAR FOLLOW-UP PERIOD

**DOI:** 10.1093/geroni/igz038.2556

**Published:** 2019-11-08

**Authors:** Yoshiko L Ishioka, Midori Takayama, Ikuko Sugawara

**Affiliations:** 1 Keio University, Yokohama, Kanagawa, Japan; 2 University of Tokyo, Bunkyo, Tokyo, Japan

## Abstract

The association between activity engagement and late-life cognitive function is considered to depend on the characteristics of the activity, the cognitive processes it involves, and the life stage of participants. A better understanding of this association is required to comprehend cognitive function in old age. The present study examined the association between baseline activity engagement and cognitive changes across a 3-year period among old-old adults. We extracted data for 873 Japanese community-dwelling participants from data of the Keio-Kawasaki Aging Study. We assessed cognitive performance thrice (at baseline, 1.5-year follow-up, and 3-year follow-up) using a short version of the Mini-Mental Status Examination. For the subsequent analyses, we used three measures of cognitive function: total score, orientation, and concentration, which showed diverse individual differences. We measured the frequency of physical activity and social group participation at baseline. Using conditional latent growth curve models, we examined which baseline activity was associated with the three measures of cognitive function over 3 years. Greater physical activity was significantly related to higher rate of orientation, after adjusting for age and education (β = −.261, p < .001). Social activity was significantly related to rates of higher total cognitive score (β = −.276, p < .001) and higher orientation (β = −.207, p < .001). These findings suggest that the association between activity engagement and late-life cognitive function among old-old adults varies by activity type and cognitive domain.

